# Regulation of Kainate Receptor Subunit mRNA by Stress and Corticosteroids in the Rat Hippocampus

**DOI:** 10.1371/journal.pone.0004328

**Published:** 2009-01-30

**Authors:** Richard G. Hunter, Rudy Bellani, Erik Bloss, Ana Costa, Katharine McCarthy, Bruce S. McEwen

**Affiliations:** Laboratory of Neuroendocrinology, The Rockefeller University, New York, New York, United States of America; James Cook University, Australia

## Abstract

Kainate receptors are a class of ionotropic glutamate receptors that have a role in the modulation of glutamate release and synaptic plasticity in the hippocampal formation. Previous studies have implicated corticosteroids in the regulation of these receptors and recent clinical work has shown that polymorphisms in kainate receptor subunit genes are associated with susceptibility to major depression and response to anti-depressant treatment. In the present study we sought to examine the effects of chronic stress and corticosteroid treatments upon the expression of the mRNA of kainate receptor subunits GluR5-7 and KA1-2. Our results show that, after 7 days, adrenalectomy results in increased expression of hippocampal KA1, GluR6 and GluR7 mRNAs, an effect which is reversed by treatment with corticosterone in the case of KA1 and GluR7 and by aldosterone treatment in the case of GluR6. 21 days of chronic restraint stress (CRS) elevated the expression of the KA1 subunit, but had no effect on the expression of the other subunits. Similarly, 21 days of treatment with a moderate dose of corticosterone also increased KA1 mRNA in the dentate gyrus, whereas a high corticosterone dose has no effect. Our results suggest an interaction between hippocampal kainate receptor composition and the hypothalamic-pituitary-adrenal (HPA) axis and show a selective chronic stress induced modulation of the KA1 subunit in the dentate gyrus and CA3 that has implications for stress-induced adaptive structural plasticity.

## Introduction

The hippocampal formation, due to its high levels of expression of receptors for corticosteroid stress hormones, is particularly susceptible to weathering and structural changes as a result of chronic stress and stress related diseases such as depression. The interplay of corticosteroids and ionotropic excitatory amino acid receptors in producing structural and physiologic changes in the hippocampal formation has been the subject of a significant amount of research, but most of this research has focused upon NMDA and AMPA receptors while relatively little has sought to describe the effects of corticosteroids upon the expression of kainate receptors (KAR).

There are five members to the KAR gene family: GluR5, 6 and 7 and KA 1 and 2 [1] and kainate receptors are comprised of various admixtures of the five subunit proteins produced by these genes. The KARs contribute to both excitatory neurotransmission and the presynaptic modulation of neurotransmitter release [2]–[4]. Notably, KAR activation contributes to LTP in the hippocampus, particularly at the mossy fiber synapse of the CA3 [5].

A number of recent clinical studies have shown KARs have potentially important roles in a number of major mental disorders, particularly depression. Polymorphisms in the KA1 receptor have been associated with response to the anti-depressant citalopram and GluR6 has been associated with suicidal ideation during treatment with the same drug [6], [7]. The GluR7 gene has also been connected to recurrent major depression [8]. KA1, GluR5 and GluR6 have also shown association with schizophrenia and bipolar disorder [9], [10].

The first study to examine the effects of corticosteroids upon hippocampal KAR was performed by Clark and Cotman [11], who tested the effects of adrenalectomy and corticosterone (CORT) replacement on binding at AMPAR, KAR and NMDAR and found no replicable effect of corticosterone or adrenalectomy on ^3^H kainate binding. Watanabe [12], did however, observe a decrease in ^3^H kainate binding after adrenalectomy, an effect which was blocked by replacement with the selective mineralocorticoid receptor (MR) agonist aldosterone but not the glucocorticoid receptor (GR) selective agonist RU28362. A study examining the expression of mRNA for KAR subunits 3 days after adrenalectomy and in response to acute high and low dose CORT showed that low doses of CORT, which presumptively occupy only MR, increased expression of all subunits, while ADX or high dose CORT (occupying both MR and GR) failed to significantly alter expression of any subunit [13]. Finally, chronic peripheral administration of the GR agonist dexamethasone increases expression of GluR6 protein in the dentate gyrus and CA3 [14]. To date these studies constitute most of what is known about the interactions of corticosteroids and the KAR. The present study aims to add to our understanding of these interactions by examining the extent to which different kainate receptor subunit mRNA's are regulated differentially by stress and adrenal steroids, using adrenalectomy, hormone replacement, chronic restraint stress and chronic corticosterone treatment of adrenally intact animals.

## Results

### Effects of adrenalectomy and adrenal steroid replacement

**Figure 1:**
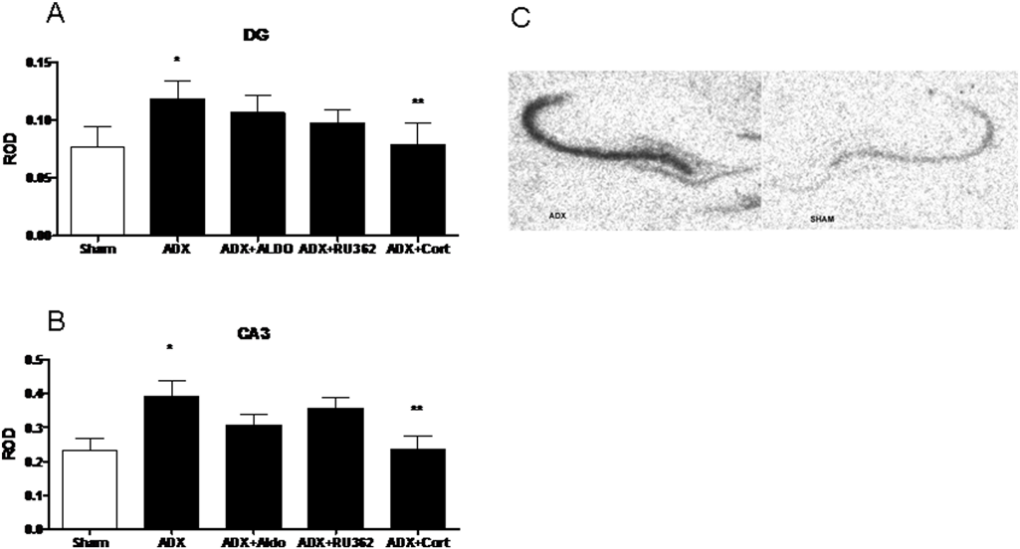
ROD of KA1 mRNA in the dentate gyrus (A) and CA3 (B) after adrenalectomy and treatment with vehicle (ADX), aldosterone (ADX+Aldo) RU28,362 (ADX+RU362) or corticosterone (ADX+CORT). (C) A representative autoradiogram of KA1 mRNA. *-significantly different from sham and ADX+Cort (p<0.05, n = 8). **-significantly different from ADX (p<0.05, n = 8).

In order to examine the effect of corticosteroids on the expression of KAR subunits, we performed *In situ* hybridization (ISH) after adrenalectomy and subacute treatment with corticosterone, aldosterone and RU28362. ISH revealed KAR subunit specific patterns of expression in the subfields of the hippocampal formation, consistent with previous reports [15]. There was a significant main effect of treatment (F (4,28) = 3.824, see Figure 1.) upon KA1 mRNA expression in the CA3 and dentate gyrus regions of the dorsal hippocampus, but no main effects were seen in the CA1 or CA2 regions. Adrenalectomy (ADX) increased KA1 message by 68±11% in the CA3 and by 54±13% in the dentate (p<0.05 versus sham, n = 8), while neither the selective MR agonist aldosterone nor the selective GR agonist RU28362, given alone, reversed this effect. However, treatment with corticosterone significantly reduced KA1 mRNA from ADX levels (p<0.05), suggesting that MR/GR heterodimers may regulate expression specifically in the dentate gyrus and downstream in the CA3.

**Figure 2:**
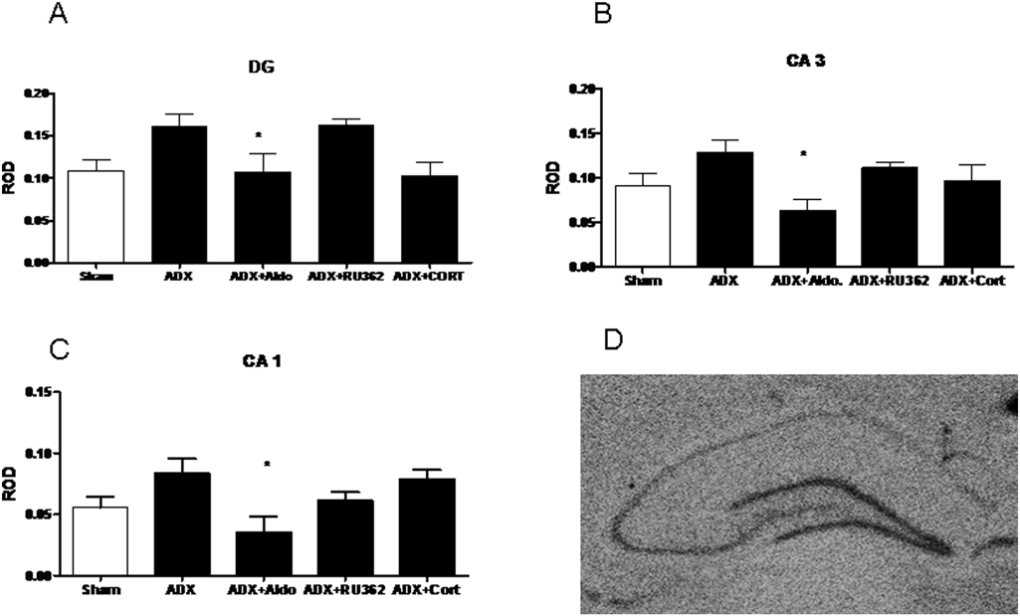
ROD of GluR6 mRNA in the dentate gyrus (A) CA3 (B) and CA1 (C) after adrenalectomy and treatment with vehicle (ADX), aldosterone (ADX+Aldo) RU28,362 (ADX+RU362) or corticosterone (ADX+CORT). (D) A representative autoradiogram of GluR6 mRNA. *-significantly different from sham and ADX+CORT (p<0.05, n = 8).

The GluR6 results, shown in Figure 2, suggest that this receptor subtype is predominately regulated via MR in the DG, CA1 and CA3. There was a main effect of treatment on GluR6 mRNA expression in the CA1 (F (4,28) = 3.778), CA3 (F (4,26) = 2.991) and dentate gyrus (F (4,26) = 4.338) after corticosteroid manipulations. In the DG, CA1 and CA3, ADX treatment showed a modest but non-significant trend toward increased GluR6 expression, and ADX+Aldosterone treatment significantly reduced mRNA compared to ADX treatment (p<0.05, n = 8), with no effect compared to sham. These data suggest that GluR6 mRNA is predominantly regulated through MR in the dentate gyrus, CA1 and CA3.

**Figure 3:**
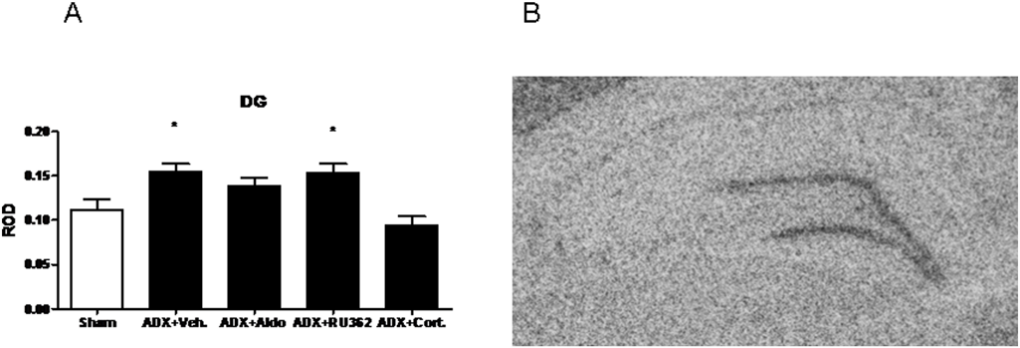
ROD of GluR7 mRNA in the dentate gyrus (A) after adrenalectomy and treatment with vehicle (ADX), aldosterone (ADX+Aldo) RU28,362 (ADX+RU362) or corticosterone (ADX+CORT). (B) A representative autoradiogram of GluR6 mRNA. *-significantly different from sham and ADX+CORT (p<0.05, n = 8).

For GluR7, there was a main effect of treatment on mRNA expression in the dentate gyrus (F (4,32) = 7.789, see Figure 3.). Relative to Sham, ADX increased expression by 39±5% (p<0.05) and ADX+RU362 increased expression by 37.9±6% (p<0.05). ADX+CORT replacement significantly reduced GluR7 message levels from both ADX+Vehicle, ADX+Aldo, and ADX+RU362.

**Figure 4:**
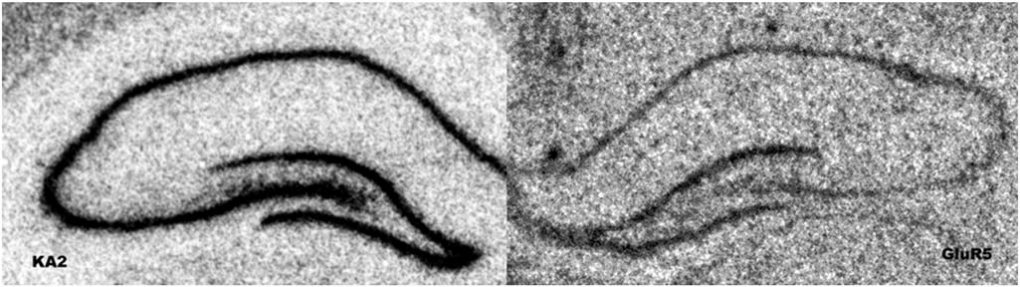
Representative photomicrographs showing KA2 mRNA signal on the right and GluR5 mRNA on the left. We did not observe changes in expression of either of these transcripts.

No main effects of treatment were observed with either GluR5 or KA2 after chronic corticosteroid manipulations (see Figure 4. for representative autoradiograms of KA2 and GluR5 expression in the hippocampal formation) .

### Effects of chronic restraint stress

**Figure 5:**
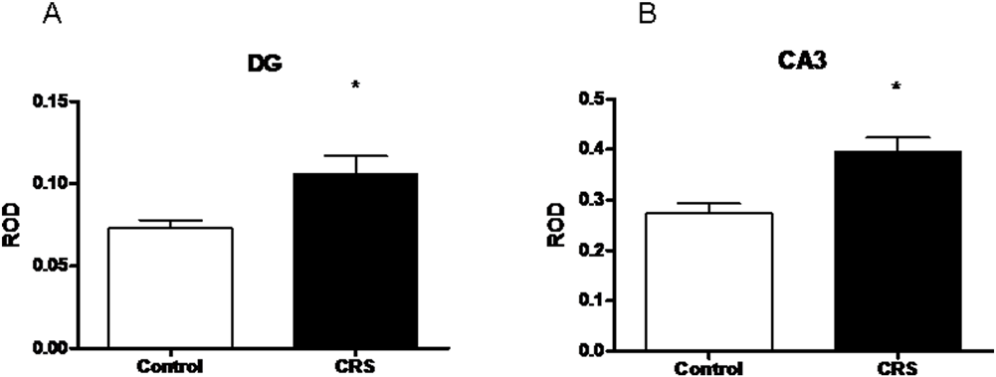
ROD of KA1 mRNA in the dentate gyrus (A) and CA3 (B) after CRS. *-significantly different from unstressed controls (p<0.05, n = 8).

To examine the effects of a stress paradigm known to cause structural and functional changes in the hippocampus on KAR expression, KAR subunit levels were measured in the hippocampus in response to 21-day chronic restraint stress (CRS). Spironolactone, an MR antagonist, was used concurrently to assess the possible contribution of MR to any stress effect. As can be seen in Figure 5, a main effect of treatment was observed on KA1 subunit expression in both the CA3 (F (2,21) = 7.817) and DG (F(2,21) = 4.285). In the CA3, CRS and CRS+Spironolactone significantly elevated expression compared to control. In the DG, a similar increase was seen with CRS, but not with CRS+Spironolactone. No other effects of CRS or CRS+Spironolactone were seen with KA2, GluR5, GluR6, or GluR7. Final body weights of both stressed groups (393.4±7.3g for CRS alone and 372.4±8.1g for CRS and spironolactone) were significantly lower than controls (430.2±6.6g, p<0.05), confirming that CRS was effective systemically.

### Effects of chronic corticosterone in drinking water

**Figure 6:**
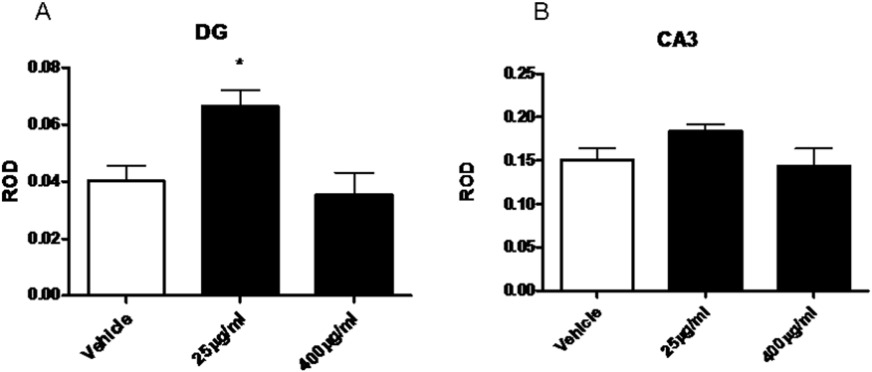
ROD of KA1 mRNA in the dentate gyrus (A) and CA3 (B) after 21 day treatment with either vehicle, 25 µg/ml or 400 µg/ml corticosterone in drinking water. *-significantly different from vehicle treated animals (p<0.05, n = 8).

To confirm that the effects of CRS were corticosteroid dependent, we treated rats for 21 days with vehicle, 25 µg/ml or 400 µg/ml corticosterone. As shown in Figure 6, treatment with a moderate dose of 25 µg/ml, but not a high dose of 400 µg/ml of corticosterone significantly (p<0.05) increased KA1 mRNA levels in the dentate gyrus (F (2,16) = 6.504) but did not reach significance in the CA3.

### Apoptosis in the dentate gyrus

Adrenalectomy produced a 46% (p<0.00001) increase (from 3.2 to 4.8% of total cell profiles) in the number of pyknotic cells in the dentate gyrus relative to sham adrenalectomized animals (data not shown).

## Discussion

Our studies reveal a complex pattern of changes in kainate receptor subunit expression induced by adrenalectomy and corticosteroid replacement and a significant and somewhat paradoxical effect of CRS and chronic corticosterone on KA1 mRNA levels, but no effect of CRS upon either KA2 mRNA levels or levels of GluR5-7 mRNA. This pattern, which was found in the dentate gyrus (DG) and CA3 region of the hippocampal formation, demonstrates that CRS involves more than adrenal steroid mediation and that increased KA1 mRNA levels may help explain morphological changes caused by CRS in the DG and CA3. Moreover, the results for KA1 mRNA levels highlight the potential of adrenal steroids to oppose certain actions of stress, which is analogous to their ability to inhibit inflammatory cytokine production.

### Effects of adrenalectomy and steroid replacement

The use of chronic adrenalectomy might have potentially confounded the interpretation of our results as adrenalectomy can produce apoptosis in dentate granule cells [16]–[18]. In our experiment, adrenalectomy did increase the number of pyknotic cells observed in the dentate gyrus, though the total percentage of pyknotic cells was never higher than 5%. While we cannot exclude dentate apoptosis as the reason for the change in KAR mRNA levels we observe in that region, it seems an improbable explanation for a number of reasons. First, the changes in mRNA levels we observed after adrenalectomy were generally increases. Further, the changes we saw in the dentate were mirrored in the CA3 (GluR6 and KA1) and CA1 (KA1), suggesting that in these cases at least, the change is more likely due to a direct effect of our manipulations of steroid levels, rather than an indirect one due to cell death in the dentate gyrus.

GluR6 mRNA levels increased after adrenalectomy in all regions of the hippocampus examined. This effect was reversed by aldosterone treatment, but not by the specific glucocorticoid receptor agonist, RU28362. This implicates the MR in the control of GluR6 mRNA levels in the hippocampal formation. Joels [13], also observed a non-significant increase in GluR6 in the DG after 3 days of adrenalectomy. Collectively, these observations suggest that MR activation inhibits GluR6 expression within the hippocampal formation.

KA1, but not KA2, mRNA expression also increased after 7 days of adrenalectomy, but the effect was reversed by high dose corticosterone rather than either the MR or GR selective agonists. GluR7 mRNA expression showed a similar pattern to KA1. We observed no changes in KA2 or GluR5 though expression of the latter was very low, which may have limited our ability to detect subtle changes. That KA1 and GluR7 were regulated by corticosterone but not by selective GR or MR agonists suggests they may be regulated by MR/GR heterodimers, a permutation of classical steroid receptor signaling recently described in cell culture [19], but as yet undescribed in vivo.

### Effects of chronic restraint stress

KA1 expression also increased after CRS; in fact, it was the only KAR subunit to do so. This is interesting because KA1, in contrast to KA2, appears to have a largely pre-synaptic localization at the mossy fiber synapse [20]. Presynaptic KARs have been shown to act as facilitating autoreceptors at the mossy fiber synapse [2], [21]–[23].

These findings, therefore, suggest a potential mechanism for the increase in hippocampal glutamate levels observed after stress [24], [25], namely, that they mediate a feed-forward enhancement of glutamate release from mossy fiber terminals. Mossy fiber activation by glutamate has been identified as a key factor in the damaging effects of kainic acid on CA3 neurons [26]–[28].

### Effects of chronic corticosterone treatment

Similarly to the effects of CRS, chronic treatment with a moderate dose of corticosterone produced an elevation of KA1 mRNA in the dentate, similar to that produced by chronic restraint stress. In the CA3, which has comparatively little GR [29], [30], this effect was not present, suggesting that the changes observed in the CA3 with CRS are the result of other mediators of the response to chronic stress, such as increased activity of the glutamate system in the hippocampus [25] . Interestingly, the response to chronic corticosterone showed an inverted-U shaped dose response, an effect often seen with regard to the effects of glucocorticoids on brain [31]. Chronic restraint, which produces a moderate elevation of corticosterone levels similar to that produced by our low dose treatment, but not as high as those produced by the 400 µg/ml dose [32], [33] fits with this interpretation, as do the findings of Joels[13], who also found that KA1 mRNA expression was enhanced more by a lower dose of cort than by a high dose. our results suggest that KA1 is also subject to regulation by corticosteroids in an inverted U shaped fashion.

### Adrenal steroids oppose effects of CRS in CA3 and dentate gyrus

The role of adrenal steroids, at least based on the effects of adrenalectomy and hormone replacement reported in this study, is somewhat paradoxical and not unlike their anti-inflammatory effects [34]. Moreover, the observation of increased KA1 expression in both adrenalectomy and CRS, however, is similar to what has been observed for the glutamate transporter, GLT-1, namely, an increased expression of GLT-1 after CRS but also an increase after ADX that is reversed by adrenal steroid replacement [35], [36]. It is possible, for both GLT-1 and KA1, that two different processes are operating in the two different treatment schemes.

One may speculate that, under basal conditions, adrenal steroids may help to maintain the basal level of kainate receptors, as well as GLT-1, so as to *homeostatically* regulate the level of glutamate release and glutamatergic activity. According to the present study, this type of regulation also applies to GluR6 and GluR7, but not to GluR5 or KA2 mRNA expression. Yet, at the same time, acute restraint stress elevates extracellular glutamate levels, measured by microdialysis, and these elevations are blocked by adrenalectomy [24]. Moreover, we show in the present study that CRS produces a feed forward, *allostatic* up-regulation of the KA1 subunit that may contribute to the dendritic retraction caused by CRS, which is mediated in part by excitatory amino acids [37]. Finally, our finding that moderate doses of CORT in the drinking water mimic the CRS induced increase of KA1 mRNA levels whereas high oral doses of CORT fail to elevate KA1mRNA indicates that a hormetic inverted U shaped dose response is operating [38]. Moreover, this hormetic dose response relationship may help explain the paradoxical finding that, while both CRS and chronic CORT each separately cause shrinkage of dendrites of CA3 neurons via a process dependent on glutamate release, the combination of CRS plus chronic CORT treatment, which presumptively elevates CORT levels beyond those produced by either treatment alone, prevented the dendritic remodeling [39].

Future work, when specific antibodies become available, needs to determine whether this up-regulation at the mRNA level is reflected in increased KA1 protein expression (as subunit specific radioligands are as yet unavailable), as well as determine the extent to which stress-induced glucocorticoid secretion may be involved in these changes. Examination of the behavior of KARs after chronic stress using electrophysiology might also provide us with a window on the functional role of these receptors in the adaptation of the hippocampus to stress, although this approach may also be impaired by the lack of selective drugs. Another important question to answer will be the extent to which chronic stress or corticosteroid treatment alters the response of KARs to and acute stressor or corticosteroid treatment, as this will allow us to begin to assess the extent to which KARs are involved in resilience to stress versus stress induced pathophysiology.

These findings are made more interesting by recent findings associating KA1 and GluR6 and 7 with major depression and other major mental disorders [6]–[10], all the more so because the subunit which definitively did not change expression levels in our experiments, KA2, has thus far shown no association with affective disorders either. Further understanding of these changes could permit an improved understanding of both stress induced pathologies and the reasons why these pathologies can take a substantial amount of time to reverse, as is the case with major depression.

## Methods

### Animals

Adult male Sprague-Dawley rats were obtained from Charles River Laboratories (Kingston, NY) at 70 days of age. Animals were housed 2–3 per cage (same age cage mates) in clear polycarbonate cages with wood chip bedding. All animals were maintained on a 12 h light-dark schedule (lights on at 0800 h) and the temperature was kept at 21±2°C. All animals had *ad libitum* access to food and water. All procedures were carried out in accordance with the guidelines established by the NIH Guide for the Care and Use of Laboratory Animals.

### Chronic Restraint Stress

Animals were left undisturbed after arrival for one week after delivery. Stressed animals were restrained in wire mesh restrainers, secured at the head and tail ends with large binder clips. Chronic stress was administered for 6 hours daily for 21 days from 10:00 to 16:00. Animals were returned to their home cages immediately after termination of the stressor. These animals were sacrificed by decapitation roughly 24 hours after the last stress (i.e. between 1300 and 1700 h). Brains were removed and flash frozen on dry ice and then stored at −80°C until processing.

### Steroid Treatments

These treatments follow those administered in [12] with some modification. We chose to follow the one week time period used by Watanabe for two reasons: first, he observed changes in KAR levels after one week of steroid replacement. Secondly, after adrenalectomy there is a progressive apoptosis of dentate gyrus granule cells [16] and while we have successfully detected changes in mRNA at the seven day time point in the past [12], [40], we were concerned that at later time points the potential for confounds would be much greater. Animals were anesthetized using ketamine and xylazine and the adrenal glands removed, save for one group which received a sham surgery. During the same surgery, osmotic mini-pumps (Alzet, Cupertino, CA) were implanted subcutaneously between the scapulae. These pumps delivered vehicle (50% polyethylene glycol), the mineralocorticoid receptor agonist aldosterone at 10 µg/hour or the glucocorticoid receptor agonist RU28,362 at 10 µg/hour. Animals who underwent ADX received 0.9% saline in their drinking water and one group received 400 µg/ml corticosterone in addition to the saline. Seven days after the completion of the surgeries, the animals were sacrificed by decapitation and their brains removed and frozen as described above.

### Chronic Corticosterone Treatment

Animals were provided with either 2.5% ETOH (vehicle), 25 µg/ml corticosterone or 400 µg/ml corticosterone in their home cage drinking water for a period of 21 days.

### *In Situ* Hybridization

Brain sections were cut at 20 µm on a cryostat and placed on Fisher Biotech ProbeOn Plus slides (Fisher, Pittsburgh, PA). In situ hybridization began with a tailing reaction to radioactively label the oligonucleotide probes with ^35^S. The probe sequences follow those described by [41], two probe sequences were used in a cocktail in order to improve sensitivity: KA1 5′-TCC AGA GAG GAG AAA TAG CCC GGT CTG CGT CCC ATA TGA ACT CTG -3′, 5′-CTT GTA GTT GAA CCG TAG GAT CTC AGC GAA CTC CTT GAG CAT GTC-3′; KA2 5′-TTC CAC TCG GGC CTT GGC TGG GAC CTC GAT GAT CCC ATT GAT CTG-3′, 5′-GTT CTC CAG GAT ATG GGG ACG CGC CCG AAG ACA CGG GTG AGG GTT-3′; GluR5 5′-AAA TCC CTC CGA TCC TGA GCA CT TGA GGG GAG GTC TGA GGG AGG-3′, 5′-CCC GGG TTG GTT CCA TTG GGC TTC CGC GTA AAG GAT GCT AAT GCC-3′; GluR6 5′-GGT TCC TTG CGA ATA TCC GAT CCA CAA TAA GCA GAG CAG G, 5′- GGT TCC TTG CGA ATA TCC GAT CCA CAA TAA GCA GAG CAG G-3′, 5′-ACT AAA CCT GGC TAT GAC AAA GAG CAC ACA ACT GAC ACC CAA GTA-3′; GluR7 5′-CTC AGC GTT CAT GAC CTG GGC GTT GGG GCC GTC CGC GTA CTC AAA-3′, 5′-ATT CTC CAC CAC CTC AGA GCC GGG GTT GCA GGG GTG GGC ATC ATA-3′. Processing of the slides followed methods as previously described in [42]. Anatomical locations were determined with the assistance of the atlas of Paxinos and Watson [43]. Optical density was determined using MCID 5.0 (Imaging Research, St. Catharine's, OT, Canada).

### Pyknotic Cell Counts

Numbers of pyknotic cells were assessed following the method of Frye and McCormick [44]. Sections were serial to those used for autoradiography and in situ. Slides containing these sections were processed to reveal Nissl substance beginning with a brief fixation in 4% paraformaldehyde in 0.1M PB for 15 minutes after which they were washed in distilled water three times for 2 minutes per wash. Sections were then dipped in 0.1% Cresyl Violet for 2 minutes and then dehydrated in ascending concentrations of ethanol prior to clearing in xylenes for 4 minutes. After drying, the slides were coverslipped with permount. Pyknotic cells in the granule cell layer and subgranule zone of the dentate gyrus were identified in a 100× visual field as those having a small volume, membrane blebbing, and dark condensed nucleus and chromatin.

### Statistics

Optical density measurements were analyzed by a one way ANOVA for the chronic steroid study and the chronic stress study. Significant main effects and interactions in ANOVA were further analyzed using Fisher's protected least significant difference test and Tukey's test, respectively. Differences are considered significant at p<0.05. All data are presented as mean±SEM.
